# Investigation on the Synthesis Process of Bromoisobutyryl Esterified Starch and Its Sizing Properties: Viscosity Stability, Adhesion and Film Properties

**DOI:** 10.3390/polym11121936

**Published:** 2019-11-25

**Authors:** Wei Li, Jie Wu, Zhengqiao Zhang, Lanjuan Wu, Yuhao Lu

**Affiliations:** 1 College of Textiles and Garments, Anhui Polytechnic University, Wuhu 241000, Anhui, China; 2 Hefei Safood Starch Co. Ltd., Hefei 230000, China

**Keywords:** bromoisobutyryl esterification, cornstarch, synthesis process, past stability, adhesion, film properties

## Abstract

To confirm the suitable synthesis process parameters of preparing bromoisobutyryl esterified starch (BBES), the influences of the synthesis process parameters—amount of 2-bromoisobutyryl bromide (BIBB), amount of catalyst (DMAP), reaction temperature and reaction time—upon the degree of substitution (DS) were investigated. Then, to produce a positive effect on the properties of graft copolymers of BBES prepared in the near future, a series of BBES samples were successfully prepared, and their sizing properties, such as apparent viscosity and viscosity stability, adhesion, and film properties, were examined. The BBES granules were characterized by Fourier transform infra-red spectroscopy and scanning electron microscopy. The adhesion was examined by determining the bonding forces of the sized polylactic acid (PLA) and polyester roving. The film properties were investigated in terms of tensile strength, breaking elongation, degree of crystallinity, and cross-section analysis. The results showed that a suitable synthesis process of BBES was: reaction time of 24 h, reaction temperature of 40 °C, and 0.23 in the molar ratio of 4-dimethylaminopyridine to 2-bromoisobutyryl bromide. The bromoisobutyryl esterification played the important roles in the properties of the starch, such as paste stabilities of above 85% for satisfying the requirement in the stability for sizing, improvement of the adhesion to polylactic acid and polyester fibers, and reduction of film brittleness. With rising DS, bonding forces of BBES to the fibers increased and then decreased. BBES (DS = 0.016) had the highest force and breaking elongation of the film. Considering the experimental results, BBES (DS = 0.016) showed potential in the PLA and polyester sizing, and will not lead to a negative influence on the properties of graft copolymers of BBES.

## 1. Introduction

Starch as one of the most abundantly occurring organic polymers in nature [1], and also as a renewable [2], biodegradable [3], and economical [4,5,6] raw material, has widespread industrial applications [7,8]. Starch has a general formula of (C_6_H_10_O_5_)_n_, including two types of d-glucan macromolecules, i.e., linear amylose and branched amylopectin [9,10], and consists of interconnected anhydroglucose units, each of which includes three hydroxyls and are linked together by a-d-glucosidic bonds [11]. It can be modified enzymatically, physically or chemically to meet various requirements. Granule forms of starch can remain unaffected during the chemical modification where hydroxyls are converted to other functionalities [12]. Generally, chemical modification of starch can be performed by esterification reaction [13] or grafting reaction with various monomers [14,15]. The most common method for synthesizing grafted starch copolymers is radical polymerization, but this method commonly suffers from lack of control of the graft density and length, and easily forms unattached homopolymer. Recently, controlled radical polymerization such as electron transfer atom transfer radical polymerization (ARGET ATRP) has received increasing attention [16,17,18]. The significant advantages of this graft copolymerization technique include controlled graft density and length, narrow molecular weight distribution, and the formation of no homopolymer impurities. However, there is little research on this polymerization method being applied for the grafting of starch to modify the starch. Prior to the graft copolymerization by ARGET ATRP, the starch is modified to form starch macroinitiator for further conducting the ARGET ATRP technique. Currently, one of the most commonly used reagents for modifying the starch to prepare the starch macroinitiator is 2-bromoisobutyryl bromide [2,19,20].

As is well known, starch has widespread applications in the textile field as a sizing agent and n paper making as a surface-sizing agent [21,22]. However, the use of native starches in these applications is limited due to low resistance to shear and high temperatures, as well as high tendency towards retrogradation [23]. This low resistance can result in the degradation of starch macromolecules, producing the instability of paste viscosity [24,25]. The instable viscosity can lead to the instability of size pick-up for warp sizing or surface sizing, thereby exerting an adverse effect on sizing and subsequently reducing the quality of fibrous goods. The large tendency towards retrogradation not only makes the paste microheterogeneous, thereby inducing an incomplete wetting and spreading out of the paste onto the fiber surfaces, leading to a low adhesion of starch to fibers, but also results in brittle starch film. Strong adhesion has been perceived to be a valuable behavior for starch adopted for sizing in textiles and paper-making [26]. It has become a highly important factor of analyzing the quality of starch-based sizes. Furthermore, the film covering the yarn surfaces plays an important role in protecting the warps from mechanical abrasions, thereby ensuring the smooth running of the weaving [27]. In paper-making, the film can enhance the paper flexibility. As a result, the toughness of starch film occupies a very stringent place for the quality of fibrous products. Fortunately, chemical modification of starch such as traditional graft copolymerization [28,29] can involve the alteration of the physical and chemical characteristics of native starch, thereby improving the adhesion and film properties of starch. Nevertheless, there are no applications of grafted starch prepared by controlled ARGET ATRP technique in warp sizing or paper-making. In addition, if the starch macroinitiator prepared can display a positive effect on viscosity stability, adhesion and film properties, it would be quite meaningful for ensuring the positive effect on the properties generated by the grafted starch macroinitiator. Therefore, whether starch macroinitiators show an adverse influence or a positive one should be ascertained. If the starch macroinitiator shows a positive influence, a suitable modification level also needs to be confirmed.

For example, due to the similarity with polyethylene terephthalate, easy processing as well as environment-friendliness, polylactic acid (PLA) acquired from renewable resources has been extensively explored for fiber applications [30]. Nowadays, if the PLA warps or PLA filaments are woven directly, it will produce some serious problems due to poor cohesion, loose tows, and easily occurrence of entanglement and bonding, and thus the PLA warps or PLA filaments must be sized before weaving. In addition, the sizing process of polyester warps also needs to be conducted before the weaving. Obviously, hydrophobic 2-bromoisobutyryl substituents can be introduced into the starch molecules to prepare the starch macroinitiator by starch esterification. The 2-bromoisobutyryl substituents that contain ester groups are conducive to enhancing the van der Waals force at the interfaces of starch adhesive layers with PLA fibers or polyester ones, owing to chemical similarity with the esters in PLA or polyester molecular chains; thereby, they can be expected to improve the adhesion. However, it is indubitable that excessive introduction of the hydrophobic substituents will reduce the water-dispersibility of starch and adversely affect the adhesion since the paste used in sizing warps is only a water-based adhesive. Accordingly, due to the indeterminacy of the influence on the adhesion generated by the 2-bromoisobutyryl substituents, we ought to evaluate the influence on the adhesion of starch to PLA and polyester fibers, and confirm whether it is an adverse influence or a positive one on the adhesion.

**Scheme 1:**
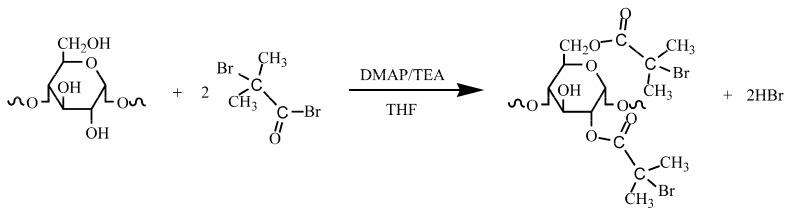
Bromoisobutyryl esterification of cornstarch for the synthesis of BBES.

Currently, there is little research about the synthesis process of starch macroinitiator, i.e., bromoisobutyryl esterified cornstarch (BBES). Therefore, an important aim of this work is to obtain suitable synthesis process parameters for preparing the BBES, as shown in Scheme 1. Moreover, there is no study that has been conducted on the properties of BBES, such as viscosity stability, adhesion and film properties. Accordingly, another objective is to reveal whether bromoisobutyryl esterification is able to enhance viscosity stability, adhesion of cornstarch to PLA and polyester fibers and film properties compared with acid-converted starch (ACS) as a control. Furthermore, the suitable level of starch modification for the BBES shall also be determined since the functional ability of a modified starch is correlated with the degree of substitution (DS) [31]. The obtained starch samples in this work were characterized by the uses of Fourier transform infra-red (FTIR) spectroscopy and Scanning electron microscopy (SEM). In addition, the measurements on DS values of BBES samples, apparent viscosity and viscosity stability of cooked starch paste, adhesion to PLA and polyester fibers, tensile strength and breaking elongation of the films, as well as film characterization with SEM and X-ray diffraction (XRD) were also performed.

## 2. Experimental

### 2.1. Materials

Natural cornstarch with a moisture content of 12.8 wt. % and an apparent viscosity of 46 mPa·s, was purchased from Shandong Hengren Industry and Trade Co., Ltd. (Shandong, China). Before use, the starch was refined to remove the protein substance [32] and subsequently acid-converted with hydrochloric acid to lower its excessive viscosity [33] to 19.5 mPa·s. Tetrahydrofuran (THF) and triethylamine (TEA) were dried with molecular sieve. 2-bromoisobutyryl bromide (BIBB) and 4-dimethylaminopyridine (DMAP) (used directly), sodium hydroxide and methanol, were purchased from Aladdin Industrial Corporation (Shanghai, China). Anhydrous ethanol was obtained from (Shanghai Maclean Biochemical Technology Co., Ltd., Shanghai, China) The pure PLA roving (520 tex) and polyester one (365 tex), adopted for adhesion measurement, were kindly supplied by BBCA Group Co. Ltd. (Bengbu, China) and Anhui Huamao Group Co., Ltd. (Anqing, China), respectively.

### 2.2. Preparation Method and Characterization of BBES for Studying Its Synthesis Process

#### 2.2.1. Preparation

The BBES was synthesized by the reaction between ACS and BIBB in a THF medium.

Briefly, dried ACS (2 g) and a certain amount of DMAP were added into a 250 mL four-necked flask connected to a mechanical stirrer, and dispersed with 100 mL of THF under mechanical agitation. Then, the TEA was added into the dispersion, and the reaction system was mechanically stirred for 30 min under N_2_ atmosphere. Subsequently, a certain amount of BIBB was added dropwise in an ice-cold bath under mechanically stirred. The reaction mixture was stirred for 1 h in the bath and heated up to a given temperature. Afterwards, the mixture was reacted at the given temperature for a certain time. Then, the final product was filtered, washed thoroughly with anhydrous ethanol, followed by freeze-dried in vacuum, powdered, and sieved with a 100-mesh sieve.

#### 2.2.2. Measurement of DS

DS indicated the number of the hydroxyls per anhydroglucose unit substituted by the 2-bromoisobutyryl substituents. Back titration with HCl preceded by alkali saponification [34,35] was employed to determine the DS. The DS value and reaction efficiency were calculated with the following Equations (1) and (2):

(1) $$\mathrm{DS}=\frac{162B}{150-150B}$$

(2) $$\mathrm{RE}=\frac{DS}{M/n}$$

where *B* and 150 denote the percentage content (%) and molecular mass of 2-bromoisobutyryl substituents, respectively, and *M* and *n* are the moles of BIBB and anhydroglucose residues of starch, respectively.

#### 2.2.3. Infrared Spectral Analysis

The Fourier transform infra-red (FTIR) spectra of ACS and BBES samples were acquired on an IRPrestige-21 FTIR Spectrometer (Shimadzu Co. Ltd., Kyoto, Japan) by using KBr disk technique to prove the successful introduction of new functional 2-bromoisobutyryl substituents in the starch molecules after bromoisobutyryl esterification. The spectra were collected in a wavenumber range of 500–4000 cm^−1^ with the running condition of 4 cm^−1^ spectral resolution.

### 2.3. Preparation Method of BBES under a Suitable Synthesis Process

Dried ACS (120 g) and anhydrous sodium sulfate (30 g) were added into a 500 mL four-necked flask connected to a mechanical stirrer and dispersed with THF to form a 30% dispersion. Then, the TEA (molar ratio of TEA to BIBB was 2:1) was added dropwise into the dispersion, and the dispersion was mechanically stirred for 60 min under N_2_ atmosphere. After the temperature of the reaction system had been cooled to 0–8 °C, certain amounts of BIBB and DMAP (dissolved with THF, respectively) were slowly added dropwise. The reaction was kept for 1 h at the above temperature and then heated up to 40 °C for 24 h. Finally, the final product was filtered, washed thoroughly with anhydrous ethanol, followed by freeze-dried in vacuum, powdered and sieved with a 100-mesh sieve.

### 2.4. Scanning Electron Microscopy (SEM) Analysis

The surface morphologies of the granular ACS and BBES samples as well as the cross-sections of their film samples were observed by means of a scanning electron microscope (Hitachi S-4800, Tokyo, Japan). Prior to SEM imaging, the samples were sprayed gold to avoid charge accumulation.

### 2.5. Apparent Viscosity and Viscosity Stability

The apparent viscosity of gelatinized starch paste (6% (*w*/*w*)) was recorded using an NDJ-79 Rotary Viscometer (Tongji Electrical Machinery Plant, Shanghai, China) at 95 °C by the method described in the literature [36]. For each paste, two individual determinations of the viscosity were conducted, and the average value was reported. Viscosity stability was determined and calculated using the method described in the work [37].

### 2.6. Adhesion Test

**Scheme 2:**
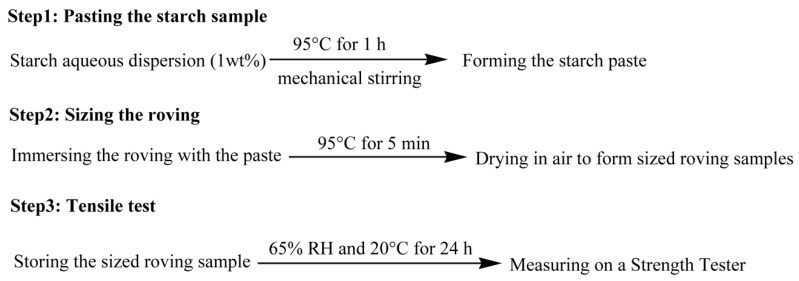
Determination of the adhesion.

Pure PLA and polyester roving were applied as adherents to examine the adhesion using the legal standard method (FZ/T 15001-2008) in China to determine the adhesion of a sizing material to fibers. The determination of the adhesion contains three steps (as shown in Scheme 2): (1) the formation of the 1% starch aqueous paste by thoroughly stirring the dispersion and heated it to 95 °C for 1 h; (2) PLA or polyester roving carefully wound onto a rectangular steel frame, was immersed in the paste for 5 min, and subsequently the immersed wet roving was atmospherically dried; (3) After storing at 65% relative humidity (RH) and 20 °C for 24 h, bonding forces of dried roving were measured on YG026D Electronic Strength Tester (Ningbo Textile Instrument Factory, Zhejiang, China) with an initial chuck-distance of 100 mm and a drawing speed of 50 mm/min at the above ambient condition [38]. For each case, the mean values of 20 successful tests were reported.

### 2.7. Surface Tension

A DCAT 21 Automatic Tensiometer (Dataphysics Co., Ltd., Filderstadt, Germany) was used to determine the surface tension of cooked starch paste. A 1 wt. % starch aqueous paste was prepared by heating the 1 wt. % starch aqueous dispersion to 95 °C and maintaining at this temperature for 1 h. After cooling down, the tension was determined in duplicate at room temperature.

### 2.8. Preparation and Measurement of Starch Films

#### 2.8.1. Preparation

The film was formed by drying cooked starch paste that was cast onto a polyester film at 65% RH and 20 °C according to the method described in our previous work [39]. Briefly, a 6% (*w*/*w*) starch aqueous paste (400 mL) was formed by preparing the starch aqueous suspension and heating the suspension to 95 °C for 1 h under mechanical agitation. Afterwards, the paste was cast onto a smooth polyester film (650 mm in length and 400 mm in width) spread on a glass plate and dried at the above ambient condition for forming the starch film.

#### 2.8.2. Measurement

Then, the films prepared were cut into 200 mm × 10 mm strips. After storing at 20 °C and 65% RH for 24 h, tensile strength and elongation at break of the films were measured on a YG026D Electronic Strength Tester in an initial chuck-distance of 100 mm and a stretching speed of 50 mm/min in accordance with the ASTM D 882-02 method. For each set of data, the averages of test results of twenty strips were reported.

### 2.9. X-Ray Diffraction (XRD) Analysis of Starch Film

The XRD patterns of ACS and BBES films were recorded on an XRD-6000 X-ray Diffractometer (Shimadzu Co., Japan) equipped with a wavelength of 0.154 nm CuKa radiation at an X-ray generator setting of 40 kV and 30 mA. The scanning region of diffraction was registered with a speed of 4°/min and a 2θ angular range of 5° to 45° with an angular step of 0.02°.

## 3. Results and Discussion

### 3.1. Process Research of Synthesizing BBES

To ascertain the suitable synthesis process of BBES, this work will mainly carry out research on the influences of the synthesis process parameters—amount of BIBB, amount of catalyst (DMAP), reaction temperature, and reaction time—upon the DS.

**Figure 1:**
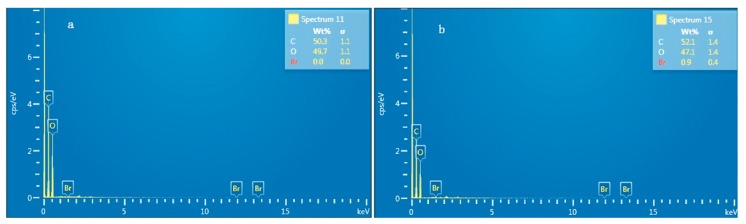
SEM-EDS images of (**a**) ACS and (**b**) BBES.

To confirm that the synthesis process of BBES in Section 2.2 could be adopted to successfully prepare the BBES samples, we performed the esterification of ACS with BIBB in a THF medium, and subsequently tested the chemical compositions of the granular product prepared by scanning electron microscopy with an energy-dispersive X-ray spectrometer (SEM-EDS) [40], as shown in Figure 1. It can be seen that the elemental composition of the product mainly comprised O, C and Br, while the ACS mainly comprised O and C. The elemental composition of Br demonstrated the successful preparation of BBES, thereby laying a foundation for subsequent research on the synthesis process of BBES.

**Figure 2:**
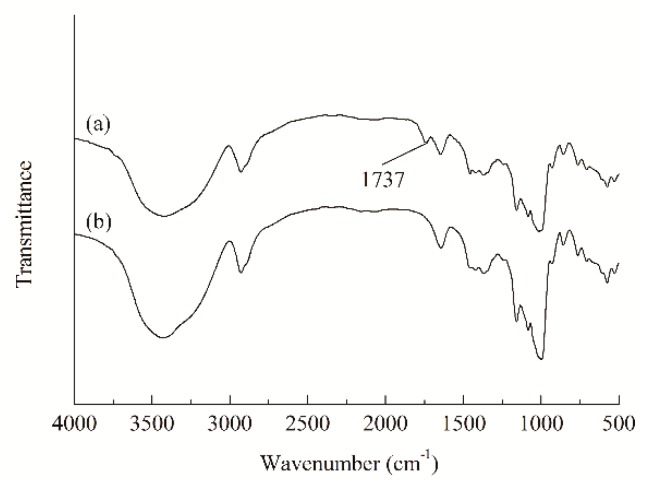
FTIR spectra of BBES with a DS value of 0.107 (**a**) and ACS (**b**).

The successful introduction of 2-bromoisobutyryl substituents into starch molecules after esterification was demonstrated by FTIR spectra of BBES and ACS, as shown in Figure 2a,b, respectively. It can be seen that there were differences in the wavenumber range of 3600–3100 cm^−1^, which were mainly attributed to the decreases of the OH groups after the esterification. In addition, the FTIR spectrum of the BBES (Figure 2a) showed an absorption bond at the wavenumber of 1737 cm^−1^, which indicated the characteristic absorption band of carbonyl groups [20,41,42] in 2-bromoisobutyryl substituents. There was no sign of this bond in the spectrum of the ACS (Figure 2b). This observation confirmed the successful introduction of 2-bromoisobutyryl substituents into the starch molecules.

#### 3.1.1. Influence of the Amount of BIBB on the DS

**Figure 3:**
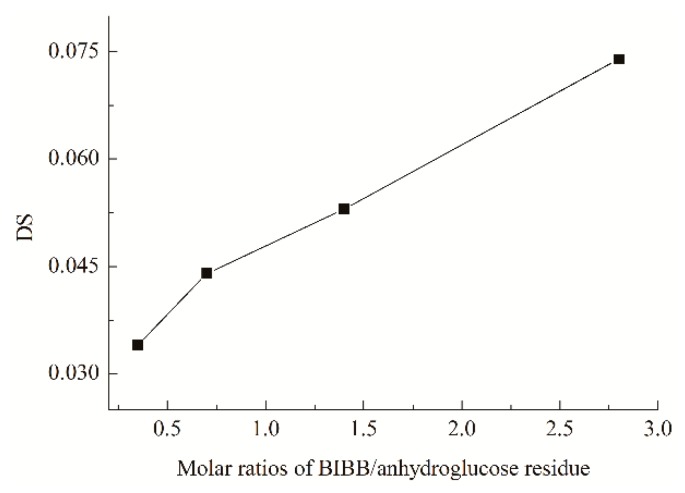
Influence of the amount of BIBB on the DS of BBES.

When the other conditions were fixed, it can be seen from Figure 3 that the DS of BBES increased as the molar ratios of BIBB to anhydroglucose residues were raised from 0.35 to 2.8. This is mainly attributed to the fact that as the amount of BIBB increases, the concentration of BIBB in the reaction system gradually increases, and the active hydroxyl groups on the macromolecular chains of ACS will have more chances to react with the BIBB, so that more hydroxyl groups in the starch molecules are esterified, thereby causing a gradual increase in the extent of esterification.

#### 3.1.2. Influence of the Amount of DMAP on the DS

**Figure 4:**
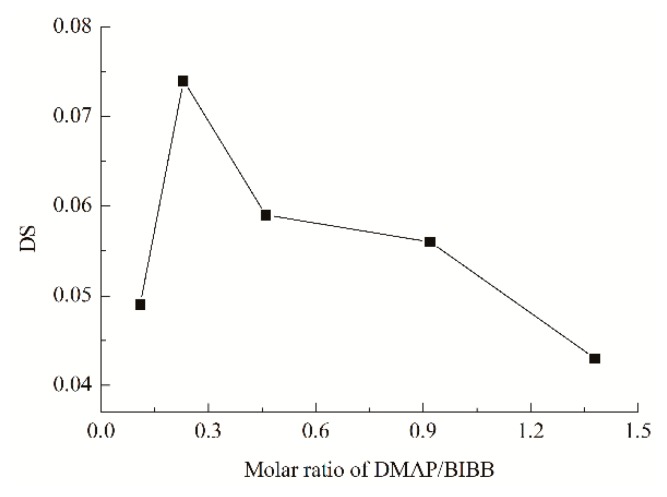
Influence of the amount of DMAP on the DS of BBES.

Under the following fixed conditions: reaction temperature of 30 °C, reaction time of 24 h, and molar ratio (BIBB/ACS) of 2.8; the influence of the amount of DMAP (denoted by the molar ratio of DMAP to BIBB) on the DS is depicted in Figure 4. As observed, when the other conditions were fixed, as the molar ratio of DMAP/BIBB was increased from 0.11 to 1.38, the DS showed a trend that first increased and then decreased. When the molar ratio was 0.23, the DS of BBES reached its maximum value of 0.074, which indicated that the most appropriate molar ratio of DMAP/BIBB was 0.23. The DS value gradually decreased once the molar ratio of DMAP/BIBB was above 0.23, which might be attributed to the fact that this might occur as a side reaction when the catalyst concentration was increased, thereby producing an adverse effect on the DS.

#### 3.1.3. Influence of Reaction Temperature on the DS

**Figure 5:**
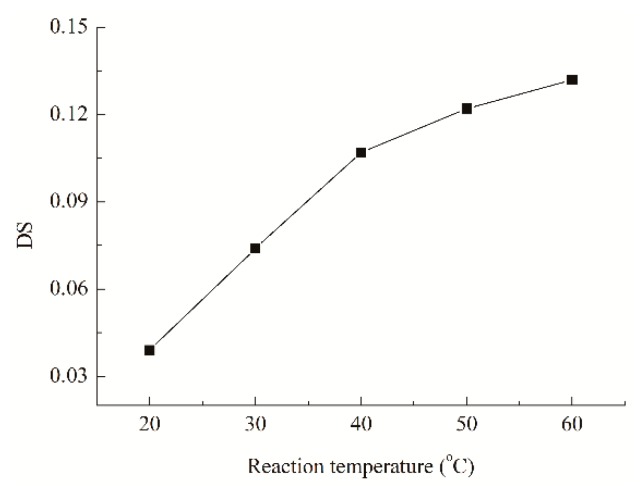
Influence of reaction temperature on the DS of BBES.

To avoid the gelatinization of starch granules in the aqueous dispersion, a reaction temperature range of 20–60 °C was selected for the study. The influence of reaction temperature on the DS is represented in Figure 5 under the following conditions: 24 h reaction time, 2.8 molar ratio of BIBB/ anhydroglucose residues, and 0.23 molar ratio of DMAP/BIBB. It can be found that the DS was dependent on the reaction temperature, and with the rise in the temperature range from 20 to 60 °C, DS showed a gradually increased tendency. With the increase in the temperature, the thermal motion of the starch macromolecules and the BIBB molecules increases, and the probability of collision between them increases, so that the DS of BBES gradually increases. However, when the temperature exceeds 40 °C, the increment in the DS lowers. This may be attributed to the fact that, as the temperature is higher than 40 °C, the excessive temperature may result in decreased activity of the DMAP. Accordingly, we concluded that the appropriate reaction temperature under the given process conditions was 40 °C.

#### 3.1.4. Influence of Reaction Time on the DS

**Figure 6:**
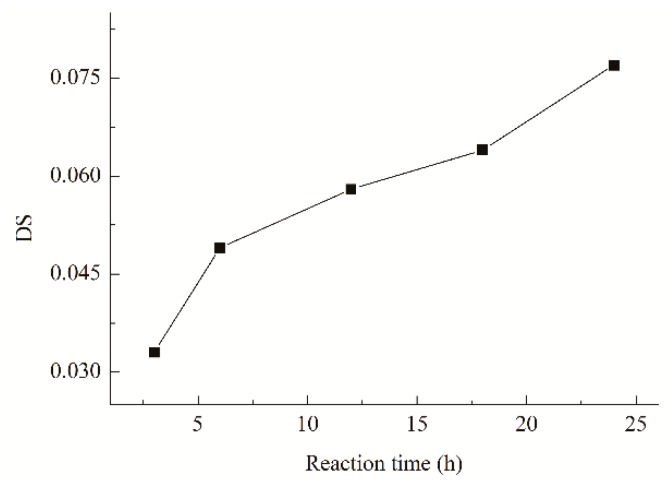
Influence of reaction time on the DS of BBES.

Figure 6 depicts the influence of reaction time on the DS under the following conditions: 30 °C reaction temperature, 2.8 molar ratio of BIBB to anhydroglucose residues, and 0.23 molar ratio of DMAP to BIBB. Under the above conditions, it can be seen that the DS of BBES exhibited a proportional relationship with the reaction time, i.e., the DS increased as raising the reaction time. In the initial stage of the reaction, BIBB reacts with DMAP to form the intermediate. After a certain reaction time, the intermediates reach a certain concentration and subsequently reacting with the hydroxyl groups on the starch chains. Therefore, with the extension of the reaction time, BIBB reacts with DMAP to form new intermediates for continuous reaction with hydroxyls, so that the DS gradually increases with the extension of the reaction time from 0 to 24 h.

### 3.2. SEM and DS Analyses of the BBES Samples with the Suitable Synthesis Process

After investigating the influences of the synthetic process parameters upon the DS, we obtain the suitable synthesis process parameters of BBES: reaction time of 24 h, reaction temperature of 40 °C, and 0.23 in molar ratio of catalyst (DMAP) to BIBB. Accordingly, to study the properties of BBES, such as adhesion-to-fibers and film properties, etc., a series of BBES samples with different DS values were prepared.

**Figure 7:**
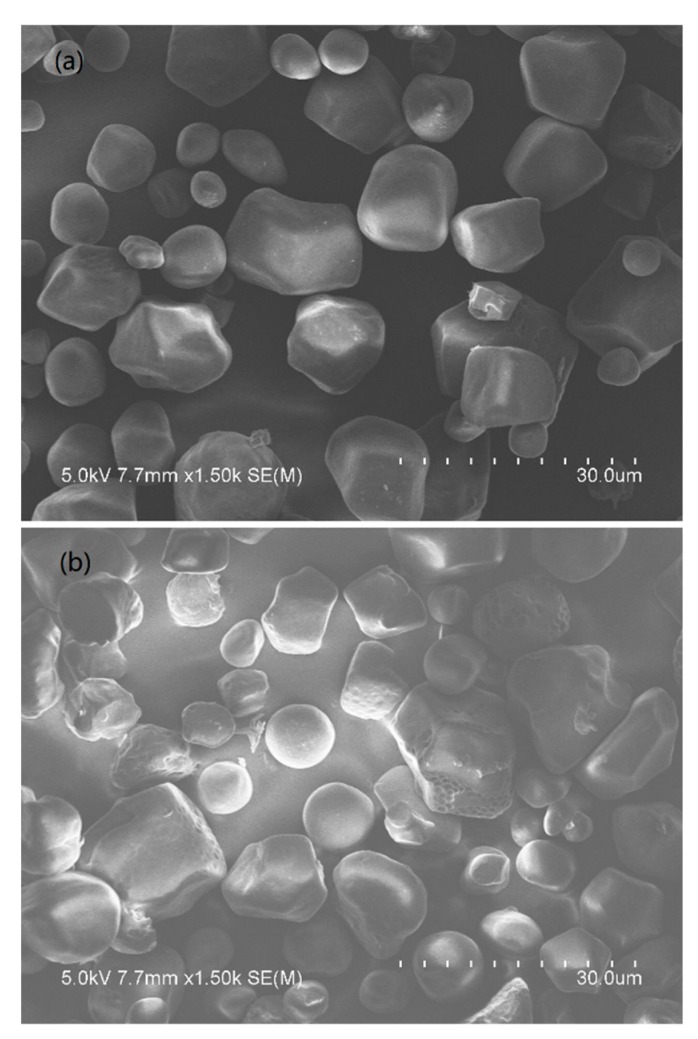
SEM images of ACS granules (**a**) and BBES ones (**b**).

SEM technique has become an important means of clearly recording the granule morphology of a modified starch [43]. Figure 7 shows the SEM images of ACS and BBES granules. As can be seen, the ACS granules were round or polygonal shapes with various sizes (Figure 7a) [44]. Nevertheless, the morphology of BBES granules was changed in comparison with the ACS. As shown in Figure 7b, the surfaces of the BBES granules underwent some visible damage, indicating the successful bromoisobutyryl esterification of granular ACS, and the modification mainly occurs on the hydroxyls of granule surfaces, as the reaction temperature is 40 °C.

**Figure 8:**
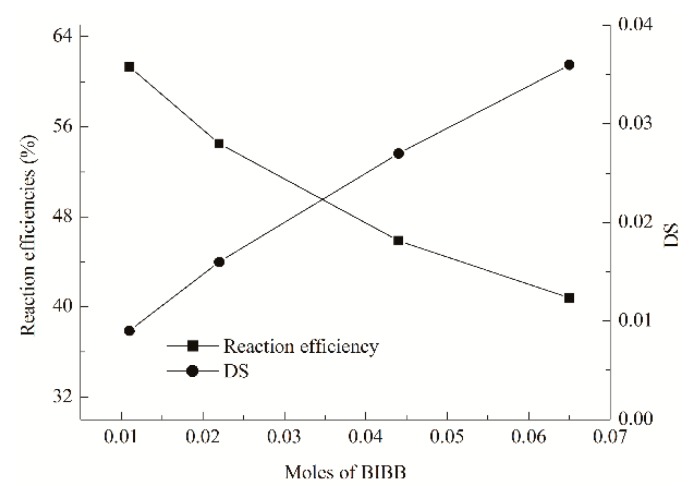
Degrees of substitution and reaction efficiencies of BBES samples prepared.

Using the suitable synthetic process parameters, the DS values and reaction efficiencies of granular BBES samples prepared were measured and denoted by plotting the DS values and reaction efficiencies versus moles of BIBB, as illustrated in Figure 8. It can be found that the DS values and reaction efficiencies were dependent on the moles of the BIBB used in the esterification, i.e., with the increases in the moles of BIBB from 0 to 0.065, the DS values gradually increased from 0 to 0.036, which indicated that BIBB with a esterification level range from 0 to 0.036 could be obtained by the reaction of ACS with BIBB in a THF medium. In addition, as the moles of BIBB increased, the efficiencies gradually decreased. As the esterification goes on, active sites are gradually occupied by the substituents introduced and there are not adequate sites for introducing new 2-bromoisobutyryl substituents into starch molecules. Consequently, the efficiencies decrease with the increase in the moles of BIBB.

### 3.3. Influence of Bromoisobutyryl Esterification

#### 3.3.1. Influence on Apparent Viscosity and Its Stability

**Figure 9:**
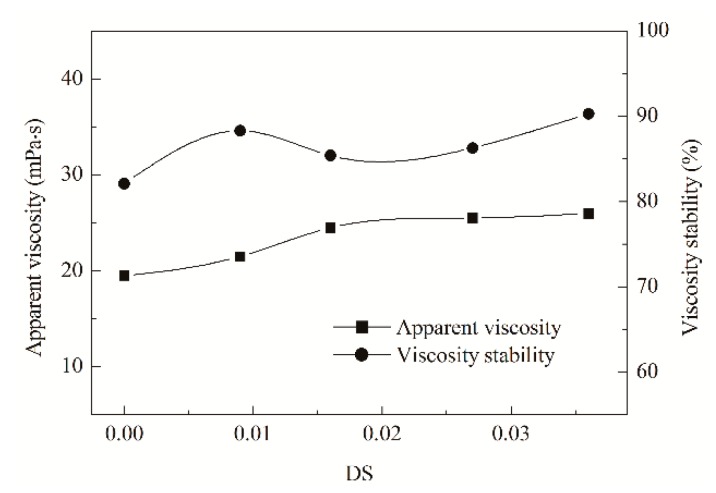
Apparent viscosity and its stability of gelatinized starch paste.

Figure 9 depicts the influence of bromoisobutyryl esterification on apparent viscosity and its stability of cooked ACS paste. As observed, the esterification was able to raise the paste viscosity compared with that of the ACS (DS = 0) paste. In addition, with the rises in DS values, the viscosities of cooked BBES pastes gradually increased. In addition, the BBES samples were superior to their counterpart ACS in stability (82.1% paste stability for ACS). As the total DS raised, the stabilities of the BBES pastes exhibited an increased tendency and were all above 85%, which fulfilled the requirement of ≥85% in paste stability for achieving a stable size pick-up during warp sizing [45]. This suggested the bromoisobutyryl esterification of ACS did not produce a negative effect on the paste stability, and the BBES paste could satisfy the requirement in the paste stability for confirming the stability of size pick-up during the sizing process.

#### 3.3.2. Influence on Adhesion

**Figure 10:**
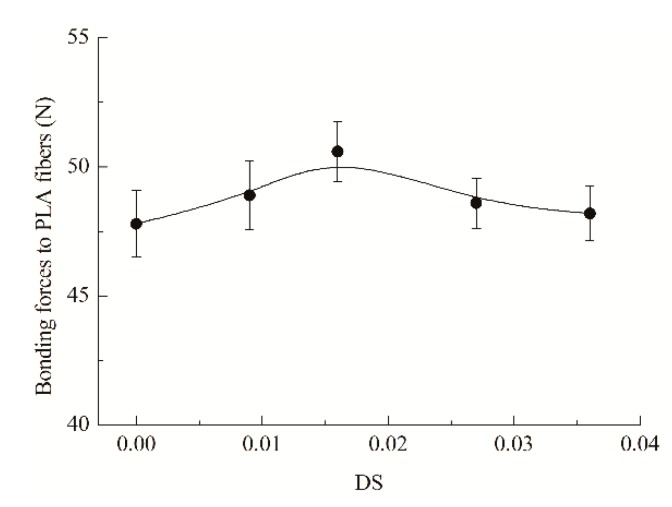
Influence of bromoisobutyryl esterification on adhesion of starch to PLA fibers.

**Figure 11:**
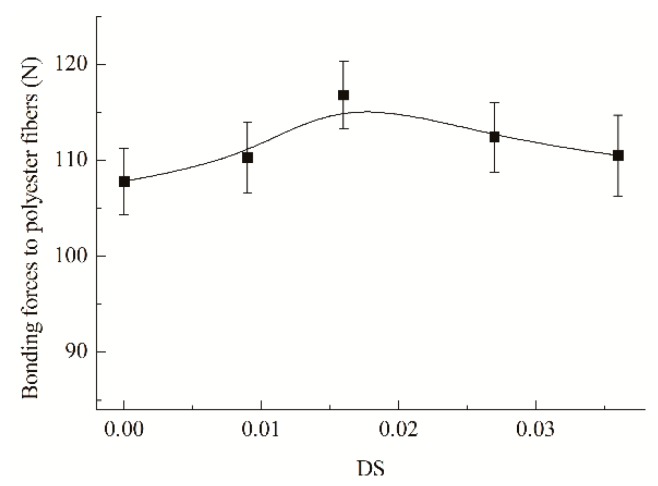
Influence of bromoisobutyryl esterification on adhesion of starch to polyester fibers.

The influence of bromoisobutyryl esterification on adhesion of ACS to PLA and polyester fibers was estimated by plotting the bonding forces versus DS values, as shown in Figure 10 and Figure 11, respectively. It can be seen that the bonding forces of ACS (DS = 0) to PLA and polyester fibers were 47.4 N and 107.8 N, respectively, and BBES was superior to ACS in the bonding forces to PLA and polyester fibers. This meant that the esterification was capable of ameliorating the adhesion of ACS to PLA and polyester fibers. The forces of BBES were correlated with the DS. With the rise in the DS, the forces of BBES to PLA and polyester fibers increased, and when the DS was 0.016, the forces reached their maximum values, at 50.6 N for PLA fibers and 116.8 N for polyester ones. When the DS was above 0.016 in the range of 0.016 to 0.036, the forces gradually decreased to 48.2 N for PLA fibers and 110.5 N for polyester ones, still showing a slight increase compared with those of ACS. This implies that bromoisobutyryl esterification will not produce an adverse effect on the adhesion of grafted BBES prepared in future based on the BBES. Based these results, the suitable DS of BBES for improving the adhesion of ACS to both fibers was 0.016.

To ascertain the influence of bromoisobutyryl esterification on adhesion, the characteristics of the gelatinized starch paste must be given attention. It is generally known that starch granules in the aqueous dispersion heated at a high temperature will swell due to adsorbing the water, and the hydrogen bonds of starch will be disrupted; sequentially the crystalline structure of the granules will be destroyed to form an amorphous structure, subsequently forming a gelatinized starch paste [46]. The paste formed can be regarded as a biphasic system, simultaneously containing disperse and continuous phases [47]. The disperse phase is swollen remnants of the granules that mainly consist of the amylopectins, and the continuous one is the solution of the soluble starch coins that is mainly comprised of linear amyloses [48]. Linear amyloses in low-temperature aqueous paste tend to orient themselves in a parallel fashion and approach each other closely enough to form aggregates [49]. Furthermore, amylose molecules are also able to produce co-crystallization with the linear branches of amylopectins [50] in the aqueous paste by the hydrogen bonding between starch hydroxyls [51]. These phenomena can lead to paste retrogradation of starch [11]. At high concentrations, the retrogradation can convert the paste into a gel that is composed of a three-dimensional network held together by hydrogen bonding [11]. It is generally accepted that a gelled paste may lose the fluidity and produce incomplete wetting and spreading of the paste onto the fiber surfaces [52]. According to the failure position, the failure of an adhesive joint commonly contains cohesive failure and interfacial one [53], which denote failures that occur wholly within the matrix of an adhesive layer formed by adhesives such as starch, and exactly at the interfaces between the layers and fibers, respectively. The interface failure and high internal stresses within the matrix of the adhesive layer [38] are prone to occur around unwetted or outspread areas, thereby generating a damage to adhesion [49].

**Figure 12:**
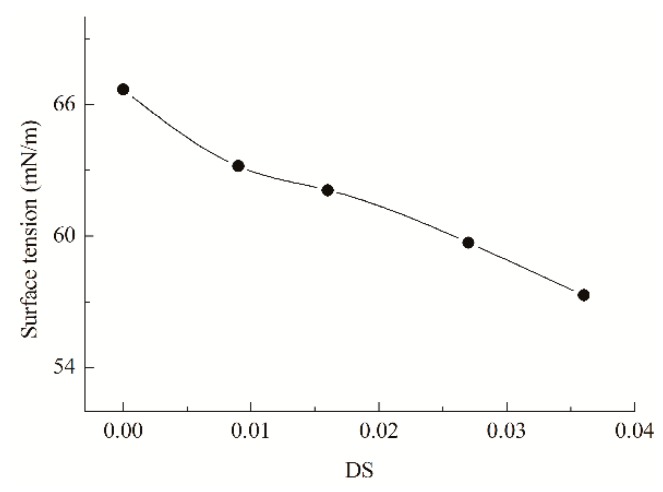
Influence of the 2-bromoisobutyryl substituents on the surface tension of cooked starch paste.

Undoubtedly, steric hindrance generated by the 2-bromoisobutyryl substituents derivatized into starch molecules can obstruct the parallel fashion and closely approach between starch amyloses in the paste, and lower the co-crystallization between the amyloses and linear branches of amylopectin, thereby favoring the diminishing of paste retrogradation. Consequently, the 2-bromoisobutyryl substituents derivatized may be expected to improve the wetting and spreading of the paste on the fiber surfaces. In addition, as broadly accepted, wetting and spreading of an adhesive liquid onto a given solid surface is closely related to surface tension [38]. A decreased tension favors wetting and spreading, and commonly induces an improved adhesion [50]. For this reason, the influence of 2-bromoisobutyryl substituents on the surface tension of cooked starch paste was investigated, as depicted by indicating the tension versus DS in Figure 12. As observed, the tension of cooked ACS (DS = 0) paste was 66.7 mN/m. The substituents had much impact on the tension, and the tension was lowered after the introduction of the substituents. The tension of the BBES paste depended on the DS, and it gradually decreased in the DS range of 0.009–0.036. This meant that 2-bromoisobutyryl substituents were necessary to provide the derivatives with stronger surface activity. A decreased tension also favors the amelioration of the wetting and spreading of the pastes onto the fiber surfaces. The amelioration will lower the probability of interfacial failure mentioned above, inducing an improvement in the adhesion. Furthermore, the hindrance is also able to raise intermolecular distance between starch chains, and reduce hydrogen bonding between starch hydroxyls, thereby exerting an internal plasticization on the matrix of starch adhesive layers. Therefore, the internal stresses within the matrix of the adhesive will be reduced after the derivatization of the 2-bromoisobutyryl substituents. Additionally, 2-bromoisobutyryl substituents contain ester groups, and display a hydrophobic characteristic, which will increase the van der Waals force at the interfaces between the layers and PLA and polyester fibers, due to the chemical similarity with the esters in PLA and polyester chains. This also favors improving the adhesion. These reasons indicate that bromoisobutyryl esterification provides a positive effect on the adhesion. Nevertheless, it is doubtless that the hydrophobic 2-bromoisobutyryl substituents derivatized can lower the water-dispersibility of starch. It has been verified that the worse water-dispersibility can cause incomplete wetting and spreading [54], thereby leading to a negative influence on the adhesion. Consequently, the combination of the positive and negative effects may be expected to reduce the probability of interfacial failure and internal stresses, favoring the adhesion. In addition, due to the combination effects, the adhesion of BBES to PLA and polyester fibers presents a trend that first increases and then decreases with increasing esterification level.

#### 3.3.3. Influence on Film Properties

During the sizing process, a portion of starch paste forms a starch film around the surfaces of sized yarns [55]. The film can provide a protective effect for the warps from mechanical abrasions, and thus enhancing their weavability [27]. Therefore, desirable film must be flexible and stretchable [27] for withstanding repeated and extensive drawing, impacting, friction, and bending actions in weaving [56]. As a result, the tensile properties of starch film were determined for evaluating the protection against the actions.

**Figure 13:**
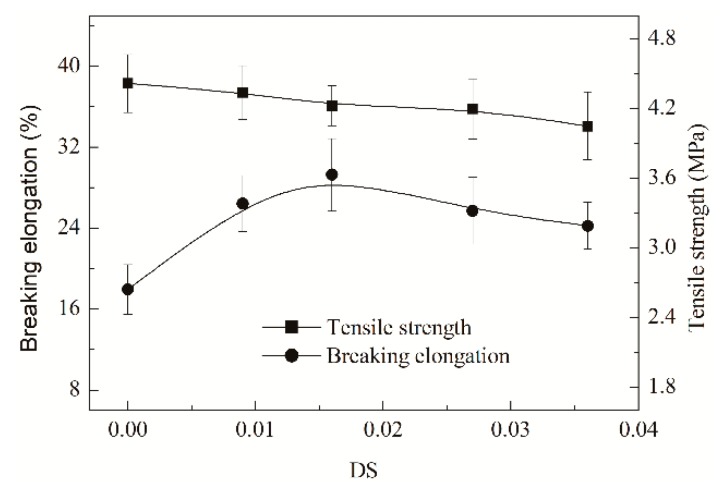
Influence of bromoisobutyryl esterification on tensile strength and breaking elongation of ACS films.

The influence of bromoisobutyryl esterification on tensile strength and breaking elongation of ACS film is shown in Figure 13. Compared with the ACS film, the films cast from cooked BBES pastes exhibited higher breaking elongation and lower tensile strength. This implied that derivatizing 2-bromoisobutyryl substituents onto the backbones of starch can decrease the brittleness of starch film. The maximal elongation (3.63%) of the BBES film was observed at the DS value of 0.016 compared with the elongation of 2.64% for the ACS film. In addition, breaking elongation and tensile strength of the BBES films were related to the DS. With increasing DS, the elongation gradually increased, reaching its highest value when the DS was 0.016, and subsequently gradually decreasing. In addition, the strength gradually reduced as the DS increased. Therefore, the BBES (DS = 0.016) film with a higher elongation is preferable to the brittle ACS film for the applications in warp sizing and surface coating, and it will not produce an adverse effect on the film properties of grafted BBES prepared in future based on the BBES.

**Figure 14:**
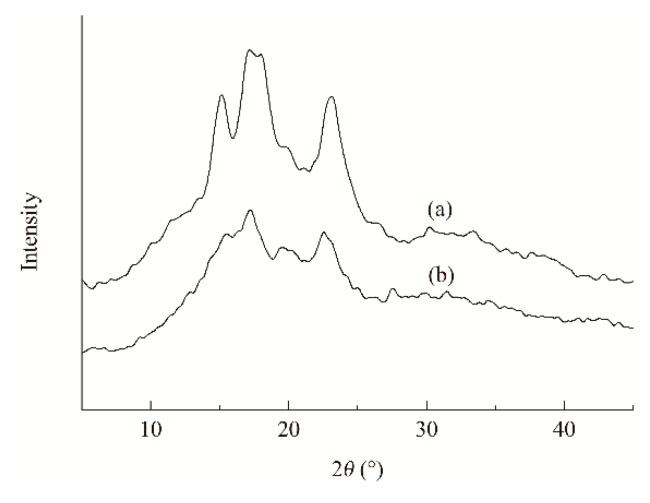
X-ray diffraction of (**a**) ACS film and (**b**) BBES (with a DS of 0.016) one.

**Figure 15:**
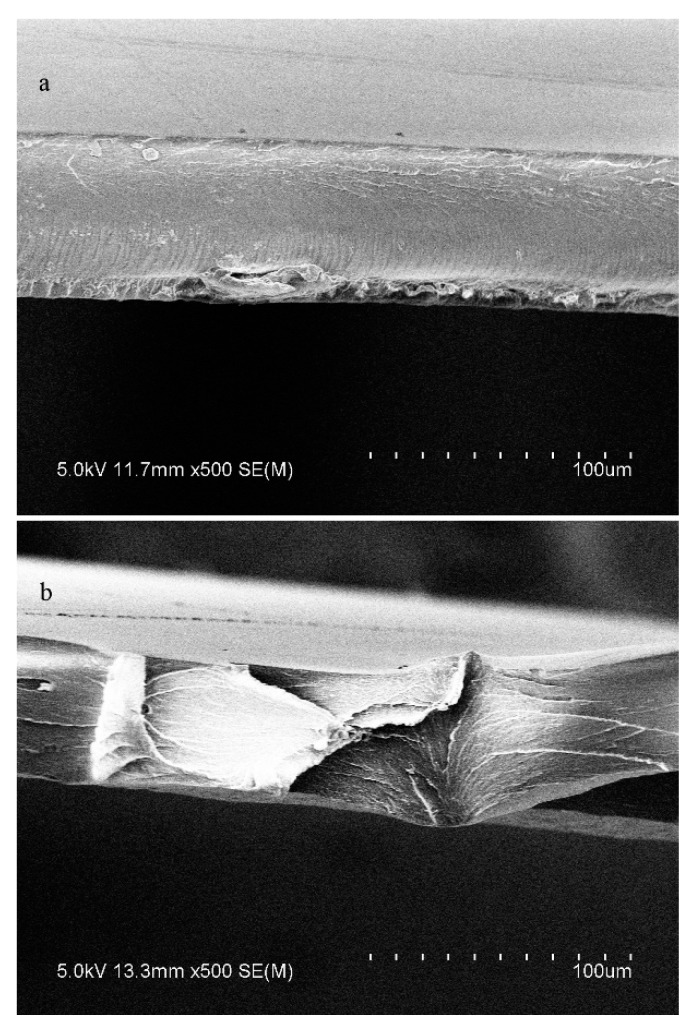
SEM images of the cross-section of ACS film (**a**) and BBES (with a DS of 0.016) one (**b**).

During formation of the film, the amyloses in aqueous paste parallelly arrange and approach closely with each other, and subsequently form a three-dimensional network structure held together by hydrogen bonding between the hydroxyls [57]. This is the main reason starch film shows the characteristics of strong brittleness and small deformation [58]. Undoubtedly, the steric hindrance of 2-bromoisobutyryl substituents favors blocking the arrangement in an orderly way of the amyloses during film formation and preventing the growth of crystal on nucleus. As a consequence, it may be expected to lower the degree of crystallinity ascertained by X-ray diffraction of the starch film, as depicted Figure 14. By comparing the X-ray diffraction patterns of the ACS film (Figure 14a) and BBES film (Figure 14b), we found that the intensity of the peaks for BBES film was lower than the intensity for the ACS one, although the location of the peaks was almost the same. The reduction of the intensity of the starch crystal peak showed that the substituents introduced lowered the formation of the crystalline structure of starch film, indicating that BBES film had a lower crystallinity (14.6%) than ACS film (21.7%). As a result, a high elongation and a low strength are exhibited for the BBES film by diminishing intermolecular hydrogen bonding compared with ACS film. This meant that the bromoisobutyryl esterification was able to lower the brittleness of starch film, providing a toughening effect for the film. The reduced brittleness provided by the esterification can be confirmed by observing the SEM images of the cross-section of ACS and BBES film samples, as shown in Figure 15. It can be observed from Figure 15a that ACS film exhibits a strong brittleness, while BBES film represents a lower brittleness, as can be seen from Figure 15b, confirming that the bromoisobutyryl esterification produced the effect of lowering the brittleness of the film.

## 4. Conclusions

Through the investigation to the influences of the synthesis process parameters—amount of BIBB, amount of catalyst (DMAP), reaction temperature and reaction time—upon the DS, we obtain suitable synthesis process parameters for BBES: reaction time of 24 h, reaction temperature of 40 °C, and 0.23 in molar ratio of DMAP to BIBB. Then, to study the properties of BBES such as adhesion-to-fibers and film properties, etc., for producing a positive effect on the properties of grafted BBES prepared in the near future, a series of BBES samples with the DS values of 0.009, 0.016, 0.027 and 0.036 and reaction efficiencies of 61.3%, 54.5%, 46.0% and 40.9%, were prepared according to the suitable synthesis process parameters. The successful introduction of the 2-bromoisobutyryl substituents was demonstrated by FTIR technique. It could be concluded that introducing 2-bromoisobutyryl substituents into starch molecules via bromoisobutyryl esterification of ACS with BIBB generating the positive effects on the properties of ACS, such as paste stabilities of above 85%, improvement in the adhesion of ACS to PLA and polyester fibers, and reduction of film brittleness. The stabilities of above 85% indicated that the introduction of the substituents could meet the requirement in the stability for warp sizing. The adhesion of BBES to both fibers was correlated with the DS, and the forces presented a trend that first increased and then decreased as increasing DS. When the DS was 0.016, the highest force was obtained. The breaking elongation of the BBES film was higher than the ACS film, and its tensile strength and degree of crystallinity were inferior to those of ACS film, indicating that 2-bromoisobutyryl substituents derivatized could lower the film brittleness (was verified by the SEM analysis of the cross-sections of ACS and BBES film samples). Considering the results of BBES with respect to paste stability, adhesion, and film properties, BBES with a DS of 0.016 showed potential in the applications of PLA and polyester sizing, and will not produce an adverse effect on the properties of grafted BBES prepared in the near future based on the BBES.

## Abbreviations

degree of substitution	DS
bromoisobutyryl esterified cornstarch	BBES
Scanning electron microscopy	SEM
polylactic acid	PLA
4-dimethylaminopyridine	DMAP
2-bromoisobutyryl bromide	BIBB
acid-converted starch	ACS
electron transfer atom transfer radical polymerization	ARGET ATRP
Fourier transform infra-red	FTIR
X-ray diffraction	XRD
tetrahydrofuran	THF
triethylamine	TEA
relative humidity	RH
scanning electron microscopy with an energy-dispersive X-ray spectrometer	SEM-EDS
